# Ethnic inequalities in older adults bowel cancer awareness: findings from a community survey conducted in an ethnically diverse region in England

**DOI:** 10.1186/s12889-021-10536-y

**Published:** 2021-03-16

**Authors:** Robert S. Kerrison, Andrew Prentice, Sarah Marshall, Sameer Choglay, Michael Levitan, Marsha Alter, Alex Ghanouni, Lesley McGregor, Christian von Wagner

**Affiliations:** 1 Research Department of Behavioural Science and Health, University College London, London, WC1E 7HB UK; 2 St Mark’s Bowel Cancer Screening Centre, St Mark’s Hospital, Watford Road, Harrow, UK; 3 Public Health England, Skipton House, London, UK; 4 The Middlesex Pharmaceutical Group of Local Pharmaceutical Committees, London, UK; 5 Division of Psychology, University of Stirling, Stirling, UK

**Keywords:** Cancer, Screening, Pharmacy, Inequalities, Ethnicity

## Abstract

**Background:**

To date, research exploring the public’s awareness of bowel cancer has taken place with predominantly white populations. To enhance our understanding of how bowel cancer awareness varies between ethnic groups, and inform the development of targeted interventions, we conducted a questionnaire study across three ethnically diverse regions in Greater London, England.

**Methods:**

Data were collected using an adapted version of the bowel cancer awareness measure. Eligible adults were individuals, aged 60+ years, who were eligible for screening. Participants were recruited and surveyed, verbally, by staff working at 40 community pharmacies in Northwest London, the Harrow Somali association, and St. Mark’s Bowel Cancer Screening Centre. Associations between risk factor, symptom and screening awareness scores and ethnicity were assessed using multivariate regression.

**Results:**

1013 adults, aged 60+ years, completed the questionnaire; half were of a Black, Asian or Minority ethnic group background (*n* = 507; 50.0%). Participants recognised a mean average of 4.27 of 9 symptoms and 3.99 of 10 risk factors. Symptom awareness was significantly lower among all ethnic minority groups (all *p*’s < 0.05), while risk factor awareness was lower for Afro-Caribbean and Somali adults, specifically (both *p*’s < 0.05). One in three adults (*n* = 722; 29.7%) did not know there is a Bowel Cancer Screening Programme. Bowel screening awareness was particularly low among Afro-Caribbean and Somali adults (both *p*’s < 0.05).

**Conclusion:**

Awareness of bowel cancer symptoms, risk factors and screening varies by ethnicity. Interventions should be targeted towards specific groups for whom awareness of screening and risk factors is low.

**Supplementary Information:**

The online version contains supplementary material available at 10.1186/s12889-021-10536-y.

## Background

Colorectal cancer (CRC, also referred to as ‘bowel cancer’) is the fourth most common cancer and the second leading cause of cancer-related death in the United Kingdom [1]. When diagnosed early, prognosis for survival is improved, with 95% of patients diagnosed at stage I surviving five or more years, compared with 10% of patients diagnosed at stage IV [2]. Unfortunately, due to the asymptomatic nature of the disease in the early stages, the majority of CRCs are diagnosed late [3], when the prognosis for survival is generally poor.

Population screening can help diagnose CRC early, by detecting cases before symptoms develop [4]. On this basis, many countries, including the United Kingdom, offer national screening programmes for CRC [5]. Despite the wide availability of screening, however, the majority of CRCs are still diagnosed in people reporting with symptoms [6]. This is thought to be, at least in part, because of the low, socially graded, awareness and uptake of screening, which is significantly lower among people living within more socioeconomically deprived and ethnically diverse areas, as well as individuals of non-White ethnicity, specifically [7, 8].

The reasons for non-uptake of CRC Screening have been investigated in-depth and are reported to relate to an interplay of factors, including: the unpleasantness of obtaining stool samples, the invasive nature of follow up tests, and emotional barriers, such as fearful and fatalistic beliefs about the potential consequences of a CRC diagnosis [9, 10]. Many of these barriers have also been found to impede timely presentation for symptoms suggestive of CRC, which is further compounded by a lack of public awareness of these symptoms as warning signs for cancer [11–14].

Like screening participation, there are social inequalities in CRC risk factor and symptom awareness [15]. A previous study by Power and colleagues (2011) found that ‘non-white’ and ‘lower socioeconomic group’ adults, living in England, had lower risk factor, symptom and screening awareness compared to their ‘white’ and ‘higher socioeconomic group’ counterparts [15]. Unfortunately, due to small numbers in each ethnic sub-group, the authors were unable to investigate ethnic inequalities in greater detail.

Since the aforementioned study was published, several cancer awareness campaigns have been conducted in England [16], including Cancer Research UK’s ‘Be Clear on Cancer’ campaign, which aimed to raise awareness of the signs and symptoms of bowel cancer, specifically [17]. The results of this study found that, while the number of patients referred onto the 2-week-wait pathway (for bowel cancer-related symptoms) was higher three months following the campaign, compared with three months before, the proportion of ‘non-White’ patients referred onto the pathway was less following the campaign, suggesting a possible widening of ethnic inequalities [17]. Again, due to small numbers in each ethnic sub-group, and a lack of long-term follow-up data, the authors were unable to investigate ethnic inequalities in greater detail, and the long-term impact of the campaign on awareness and inequalities is not known [16].

Identifying the specific ethnic groups for which risk factor, symptom and screening awareness is particularly low, as well as specific symptoms / risk factors for which these groups lack awareness, has important implications for the development of effective interventions that can be targeted to reduce inequalities in awareness. The aim of this study, therefore, was to extend our understanding of the association between ethnicity and CRC awareness, among screening eligible adults aged 60+ years, by conducting questionnaires with an ethnically diverse sample.

## Methods

### Study design and setting

To assess people’s awareness of the risk factors, symptoms and availability of screening for CRC, we conducted a cross-sectional survey with individuals living in the ethnically diverse London Boroughs of Brent, Harrow and Hillingdon.

### Participants

Participants were adults, aged 60+ years, who were eligible for bowel cancer screening during the study period (April–June, 2019).

### Participant recruitment

Given that language can be a barrier for some Black, Asian and Minority Ethnic (BAME) group adults (e.g. first generation immigrants), and they are likely to have better access to a community pharmacy (CP) than a primary care practice (in England, 89% of the population can walk to a CP within 20 min, rising to 98% in urban areas, and 99% in areas of high deprivation – the ‘*positive pharmacy care law’*) [18], we decided to recruit participants to the study through CPs, which, in addition to being more accessible than primary care practices, tend to employ people from the local community and can speak multiple languages reflective of the local population [18].

### Recruitment and training of CP staff and other community providers

All healthcare staff, working at a CP that was part of the Middlesex Pharmaceutical Committees (MPCs), during the study period, were invited to partake in the research. Eligible staff first received an email from the head of the MPCs, inviting them to participate in the study. Those who responded indicating they were interested in helping with the study were then invited to attend one of three training sessions (one training session per borough). The training was delivered face-to-face, by the St. Mark’s Bowel Cancer Screening Centre Health Promotion Team in March 2019. The training sessions provided attendees with information on bowel cancer screening, symptoms and risk factors (providing them with the correct answers to the questions, should the survey generate discussion with the patient), as well as the aim of the study and how to deliver the questionnaire to customers. Each CP was tasked with surveying 30 participants (as a guideline: one per day, throughout the month of April), verbally, in return for £160 (CPs that collected < 30 questionnaires received £5.33 for each questionnaire collected); all of the surveys were printed in English (Appendix 1).

An additional 85 members of the public were later surveyed over the phone by a member of the Harrow Somali Association (May, 2019), who received the same training as CP staff. A further 15 questionnaires were administered in person by a member of the St Mark’s Bowel Cancer Screening Centre Health Promotion Team, during a Black African and Caribbean community group meeting (June 2019). These additional questionnaires were performed to help collect data on individuals who do not typically visit a CP.

### Data collection and data entry

Completed surveys were returned to the Health Promotion Team at St Mark’s Hospital (via freepost), where the data were entered into the study database on SPSS by the Health Improvement Principal (AP). A proportion (10%) were checked and validated by another member of the team (SC).

### Measures

Awareness of the symptoms, risk factors and available screening for bowel cancer were assessed using a modified version of the Bowel Cancer Awareness Measure (BCAM): a publicly available and scientifically validated questionnaire produced by Cancer Research UK [15]. The adapted measure included a range of questions, including 9 on awareness of warning signs, 10 on risk factors and one on awareness of the UK bowel screening programme (not previously included – Appendix 1). Respondents completing the questionnaire were asked to indicate whether each of the warning signs could be symptoms of CRC, with response options: ‘Yes’, ‘No’ and ‘Don’t know’. A correct response (‘Yes’) was given a score of ‘1’, while an incorrect response (‘No’ / ‘Don’t know’) was given a score of ‘0’, generating a scale ranging from 0 (no correct responses) to 10 (no incorrect responses). For the list of risk factors, respondents were given a 5-point Likert scale, ranging from ‘Strongly agree’ to ‘Strongly disagree’. Responses were dichotomised as ‘correct’ (1) or ‘incorrect’ (0) respectively (‘Strongly agree’ / ‘Agree’ vs. ‘Neither agree nor disagree’, ‘disagree’, ‘strongly disagree’), creating a scale ranging from 0 (no correct responses) to 9 (no incorrect responses). A single question was used to assess screening awareness: ‘is there a bowel screening programme’, with response options ‘Yes’/‘No/‘Don’t know’. Responses were dichotomised as either ‘correct’ (Yes) or ‘incorrect’ (No / Don’t know). All scoring was performed by the researchers (RK and CvW).

In addition to CRC awareness, several demographic variables were measured, including: **gender** (response options: Male, Female, Prefer not to say), **age** (response options: 60–65, 66–69, 70–75, 76+, prefer not to say [all participants were asked to confirm they were 60 years or older, before commencing the questionnaire]), **ethnicity** (response options: White British, White Irish, Any other white background, Black Caribbean, Black African, Indian, Pakistani, Bangladeshi, Chinese, Any other Asian background, White and Black Caribbean, White and Black African, White Asian, Any other mixed background, Other, Prefer not to say) and **main language** (English, Sylheti, Urdu, Cantonese, Punjabi, Other, Gujurati, Somali, Arabic, Prefer not to say). The wording for the demographic questions, and their response options, were extracted verbatim from the original B-CAM [15]; however, individuals called by the Harrow Somali Association, or who attended the Black African and Caribbean community group meeting, were asked to additionally specify their ethnicity, if they stated ‘other’.

For the purpose of the analysis (described below), ethnicity was coded as **‘White British / Irish’** (White British + White Irish), **South Asian** (Indian + Bangladeshi + Pakistani), **Any other Asian ethnicity** (Chinese + Any Other Asian Background), **Afro-Caribbean** (Black African + Black Caribbean), **Somali** (Somali), **Arab** (Arab) and **Mixed / Other** (Any Other Background + Any Other Mixed Background + Any Other White Background + White Asian + White and Black African + White and Black Caribbean), while main language was coded as English (‘English’) and ‘Any other language’ (Sylheti + Urdu + Punjabi + Gujurati + Cantonese + Somali + Arabic + Other).

### Pilot testing

The development of the B-CAM is well documented and has previously been reported, in detail, by Power and colleagues [15]. Items were originally reviewed by CRC experts (*N* = 16), who considered the interpretability, clarity and accuracy of questions. Draft versions of the measure were then assessed in cognitive interviews (*n* = 17) with members of the public using the ‘think-aloud’ method [19] to assess respondents’ comprehension of the questions (i.e. whether specific words and phrases used in the question are understood as intended by researchers). Several adjustments were then made to the questions, based on the interviews (e.g. ‘straining feeling’ was changed to ‘a feeling that your bowel does not completely empty after using the lavatory’).

### Analysis

Descriptive statistics were used to report the frequency and proportion of participants who correctly identified each risk factor and symptom, both overall and by demographic subgroup (e.g. men, women, etc.), as well as the demographic characteristics of the sample. The mean number of risk factors and symptoms identified by participants was also reported using descriptive statistics; again, both overall and by demographic subgroup. Associations between mean risk factor and symptom awareness scores and demographic variables were assessed using linear regression (separate models were produced for risk factor awareness and symptom awareness). Similarly, associations between screening awareness and demographic variables were assessed using logistic regression (the corresponding method for binary outcomes). Logistic regression was also used to assess associations between awareness of individual risk factors and warning signs and demographic variables. Associations were considered ‘statistically significant’ if the *p* value was < 0.05. All analyses were performed using SPSS statistics (Ver 25.0).

### Missing data

The number of cases with missing data (including those who responded ‘prefer not to say’) for each variable was reported using descriptive statistics. Cases with missing data for the outcome variable, or one or more of the co-variates (i.e. gender, age, ethnicity, language), were excluded from the analyses. The total number of cases included in each analysis is reported in the tables.

### Collinearity

Collinearity between the predictor variables (i.e. gender, age, ethnicity and main language) was assessed using Pearson’s correlation. As all correlations between predictor variables were < 0.7 (Appendix 2), there was no evidence for collinearity between predictor variables. To be sure, we additionally calculated the variance inflation factors for the predictor variables, all of which were < 10 (Appendix 2).

### Ethical approval

The study was performed as part of the routine service improvement strategy employed by St Mark’s Bowel Cancer Screening Centre. Completion of the Health Research Authority Decision Tool indicated that NHS Research Ethics Committee Review was not required. All data were anonymous and participants provided informed consent through completion and return of the questionnaire (the purpose of the research was explained prior to data collection). The study was carried out in accordance with Good Clinical Practice guidelines and the principles set forth in the Declaration of Helsinki.

## Results

### Recruitment of CP staff

Staff from 206 CPs in Northwest London were invited to participate in the study. In total, staff from 40 CPs (19.4%) responded to take part. Each CP collected an average of 23 questionnaires.

### Sample characteristics

In total, 1013 adults completed the questionnaire (913 through CPs, 15 through St Mark’s Hospital and 85 through the Harrow Somali Association). Participants were predominantly aged 60 to 69 years (*n* = 609; 60.1%) and spoke English (*n* = 622; 61.4%; Table 1). There was an equal mix of men (*n* = 485; 47.9%) and women (*n* = 476; 47.0%; data on gender were missing for 52, i.e. 5.1%). The ethnic distribution of the sample was as follows: 46.6% (*n* = 472) **White British / Irish**, 27.2% (*n* = 276) **South Asian**, 6.7% (68) **Afro-Caribbean**, 6.2% (*n* = 63) **Somali**, 1.6% (*n* = 16) **Arab**, 5.7% (*n* = 58) **Mixed / Other** and 5.0% (*n* = 51) **any other Asian ethnicity** (data on ethnicity were missing for 9 [0.9%]). The ethnic composition of the sample was similar to that of the general population living in Northwest London in 2018 (Office for National Statistics report that, in 2018, 41.5, 34.6, 11.2 and 12.7% of the population living in Brent, Harrow and Hillingdon identified as White [i.e. White British / Irish], Asian [South Asian + any other Asian ethnicity], Black [Afro-Caribbean] and Mixed/Other [Mixed/Other + Arab + Somali], respectively) [20].

**Table 1:** Sample Characteristics (*n* = 1013)

Characteristic	n (%)
**Sex**	
Male	485 (47.9)
Female	476 (47.0)
Missing / prefer not to say	52 (5.1)
**Age (Years)**	
60–65	351 (34.6)
66–69	258 (25.5)
70–75	242 (23.9)
76+	140 (13.8)
Missing / prefer not to say	22 (2.2)
**Ethnicity**	
White British / Irish	472 (46.6)
South Asian	276 (27.2)
Any other Asian ethnicity	51 (5.0)
Afro-Caribbean	68 (6.7)
Somali	63 (6.2)
Arab	16 (1.6)
Mixed / Other	58 (5.7)
Missing / prefer not to say	9 (0.9)
**Main Language**	
**English**	
English	622 (61.4)
Any other language	376 (37.1)
Missing / prefer not to say	15 (1.5)

### Overall symptom awareness

Participants were able to correctly recognise a mean average of 4.27 (out of 9) warning signs. After adjusting for co-variates (i.e. gender, age, language), awareness was statistically significantly lower for every ethnic minority group (except ‘mixed / other’: *p =* 0.627) compared with White British / Irish (all *p*’s < 0.05; Table 2). People whose main language was not English correctly identified fewer symptoms on average than people whose first language was English (participants correctly identified 3.34 and 4.89 symptoms, respectively; *p* < 0.0001), independent of co-variates (i.e. gender, age, ethnicity). There were no statistically significant differences based on age or gender (both *p*’s < 0.05).

**Table 2:** Predictors of risk factor, symptom and screening knowledge: results from the linear regression (risk factors and symptoms) and binary logistic regression (Screening) analyses

Characteristic	Symptom score (multi-item scale) (*n* = 912)			Risk factor score (multi-item scale) (*n* = 879)			Screening score (single-item scale) (*n* = 937)		
	Mean (0–9)	***β coefficient*** Unadjusted [*p* Value]	***β coefficient*** Adjusted [*p* Value]	Mean (0–10)	***β coefficient*** Unadjusted [*p* value]	***β coefficient*** Adjusted [*p* Value]	Mean (0–1)	Unadjusted OR [*p* Value]	Adjusted OR (95%CIs) [*p* Value]
**Total**									
Total	4.27	–	–	3.99	–	–	0.72	–	–
**Gender**									
Male	4.14	Ref	Ref	3.97	Ref	Ref	0.71	Ref	Ref
Female	4.38	0.04 [0.203]	0.04 [0.171]	4.00	0.01 [0.843]	< 0.01 [0.974]	0.74	1.15 (0.87, 1.53) [0.330]	0.95 (0.69, 1.32) [0.771]
**Age (Years)**									
60–65	4.38	Ref	Ref	3.81	Ref	Ref	0.69	Ref	Ref
66–69	4.29	−0.1 [0.737]	−0.02 [0.492]	4.34	0.09 **[0.027]**	0.12 [**0.007]**	0.75	1.33 (0.93, 1.92) [0.120]	1.21 (0.80, 1.84) [0.364]
70–75	4.29	−0.02 [0.726]	−0.18 [0.659]	4.10	0.05 [0.249]	0.075 [0.085]	0.81	1.94 (1.30, 2.89) [**0.001**]	1.70 (1.08, 2.70) **[0.023]**
76+	4.16	−0.03 [0.467]	−0.05 [0.256]	3.57	0.04 [0.410]	−0.04 [0.362]	0.61	0.69 (0.46, 1.04) [0.074]	0.54 (0.34, 0.85) **[0.009]**
**Ethnicity**									
White British / Irish	5.17	Ref	Ref	4.29	Ref	Ref	0.81	Ref	Ref
South Asian	3.27	−0.25 **[< 0.0001]**	−0.28 **[< 0.0001]**	4.00	− 0.05 [0.200]	− 0.13 [0.083]	0.77	0.78 (0.55, 1.14) [0.200]	0.87 (0.48, 1.57) [0.634]
Any other Asian ethnicity	3.25	−0.21 **[< 0.0001]**	− 0.24 **[0.009]**	3.92	− 0.04 [0.388]	− 0.11 [0.252]	0.71	0.56 (0.29, 1.07) [0.079]	0.67 (0.30, 1.52) [0.339]
Afro-Caribbean	3.39	−0.21 **[< 0.0001]**	− 0.17 **[< 0.0001]**	3.45	0.09 [0.420]	− 010 **[0.043]**	0.63	0.40 (0.23, 0.69) [**0.001**]	0.38 (0.21, 0.70) **[0.002]**
Somali	2.35	−0.34 **[< 0.0001]**	− 0.36 **[< 0.0001]**	2.46	0.22 **[< 0.0001]**	0.23 **[< 0.0001]**	0.14	0.04 (0.02, 0.08) [**< 0.0001**]	0.05 (0.02, 0.12) **< 0.0001**
Arab	1.94	−0.22 **[< 0.0001]**	− 0.18 **[< 0.0001]**	2.44	− 0.12 **[0.010]**	− 0.10 [0.054]	0.00	0.00 (0.00, i) [0.998]	0.00 (0.00, i) [0.998]
Mixed / Other	4.20	−0.11 **[0.012]**	−0.03 [0.627]	4.26	−0.00 [0.946]	0.02 [0.689]	0.72	0.48 (0.26, 0.87) [0.015]	0.57 (0.28, 1.12) [0.10]
**Language**									
English	4.89	Ref	Ref	4.20	Ref	Ref	0.78	Ref	Ref
Any other language	3.34	0.26 **[< 0.0001]**	0.21 **[< 0.0001]**	3.71	−0.11 **[0.010]**	0.06 [0.141]	0.62	2.26 (1.71, 3.01) [**< 0.0001**]	1.20 (0.69, 2.11) [0.556]

### Awareness of individual symptoms

The individual symptoms for which there was the greatest awareness in the sample were: ‘Blood in stool’ (*n* = 734; 72.5%), ‘Bleeding from back passage’ (*n* = 666; 65.7%) and ‘Weight loss’ (*n* = 539; 53.2%; Table 3). Conversely, individual symptoms for which there was the lowest awareness in the sample were: ‘Pain in back passage’ (*n* = 372; 36.7%), ‘Tiredness / anaemia’ (*n* = 280; 27.6%) and ‘Bowel not feeling empty’ (*n* = 263; 26.0%).

**Table 3:** Predictors of awareness for individual symptoms: results from the binary logistic regression analyses

Symptom	Bleeding from back passage (***n*** = 940) n (%) OR (95%CI) [P]	Weight loss (n = 940) n (%) OR (95%CI) [P]	Change in bowel habits (n = 940) n (%) OR (95%CI) [P]	Pain in abdomen (***n*** = 941) n (%) OR (95%CI) [P]	Lump in abdomen (***n*** = 938) n (%) OR (95%CI) [P]	Pain in back passage (n = 938) n (%) OR (95%CI) [P]	Tiredness / anaemia (n = 940) n (%) OR (95%CI) [P]	Bowel not empty (n = 937) n (%) OR (95%CI) [P]	Blood in stool (***n*** = 937) n (%) OR (95%CI) [P]
**Total**									
Total	666 (65.7)	539 (53.2)	513 (50.6)	468 (46.2)	459 (45.3)	372 (36.7)	280 (27.6)	263 (26.0)	734 (72.5)
**Gender**									
Male	314 (64.7) Ref	245 (50.5) Ref	226 (46.6) Ref	222 (45.8) Ref	217 (44.7) Ref	184 (37.9) Ref	123 (25.4) Ref	117 (24.1) Ref	352 (72.6) Ref
Female	317 (66.6) 1.05 (0.78. 1.40) [0.764]	265 (55.7) 1.22 (0.93, 1.60) [0.152]	260 (54.6) 1.35 (1.02, 1.78) [**0.036**]	169 (35.5) 1.04 (0.80, 1.36) [0.777]	222 (46.6) 1.16 (0.89, 1.51) [0.274]	169 (35.5) 0.89 (0.68, 1.18) [0.420]	143 (30.0) 1.24 (0.93, 1.67) [0.146]	132 (27.7) 1.19 (0.89, 1.61) [0.244]	346 (72.7) 1.00 (0.73, 1.36) [0.992]
**Age (years)**									
60–65	235 (67.0) Ref	191 (54.4) Ref	181 (51.6) Ref	171 (48.7) Ref	162 (46.2) Ref	132 (37.6) Ref	99 (28.2) Ref	87 (24.8) Ref	259 (73.8) Ref
66–69	169 (55.0) 0.85 (0.58, 1.24) [0.406]	142 (55.0) 1.01 (0.71, 1.43) [0.960]	136 (52.7) 0.94 (0.66, 1.34) [0.731]	117 (45.3) 0.82 (0.58, 1.16) [0.269]	113 (43.8) 1.02 (0.72, 1.43) [0.920]	93 (36.0) 0.80 (0.56, 1.15) [0.230]	77 (29.8) 1.05 (0.73, 1.53) [0.786]	67 (26.0) 1.08 (0.74, 1.59) [0.68]	186 (72.1) 0.93 (0.63, 1.37) [0.706]
70–75	161 (66.5) 0.89 (0.60, 1.32) [0.565]	130 (53.7) 0.89 (0.62, 1.28) [0.532]	122 (50.4) 0.82 (0.57, 1.19) [0.298]	111 (45.9) 0.84 (0.59, 1.16) [0.338]	111 (45.9) 1.03 (0.73, 1.46) [0.860]	89 (36.8) 0.94 (0.66, 1.36) [0.753]	67 (27.7) 0.97 (0.66, 1.43) [0.884]	65 (26.9) 1.16 (0.79, 1.71) [0.456]	174 (71.9) 0.89 (0.60, 1.34) [0.589]
76+	90 (64.3) 0.74 (0.46, 1.17) [0.193]	68 (48.6) 0.67 (0.44, 1.03) [0.070]	65 (46.4) 0.68 (0.44, 1.05) [0.08]	64 (45.7) 0.81 (0.53, 1.23) [0.324]	67 (47.9) 1.15 (0.76, 1.75) [0.512]	54 (38.6) 0.91 (0.59, 1.41) [0.665]	33 (23.6) 0.70 (0.43, 1.14) [0.150]	41 (29.3) 1.12 (0.70, 1.78) [0.647]	103 (73.6) 0.97 (0.59, 1.58 [0.892])
**Ethnicity**									
White British / Irish	381 (80.7) Ref	312 (66.1) Ref	307 (65.0) Ref	272 (57.6) Ref	228 (48.3) Ref	220 (46.6) Ref	161 (34.1) Ref	140 (29.7) Ref	396 (83.9) Ref
South Asian	164 (59.4) 0.44 (0.26, 0.74) [**0.002**]	125 (45.3) 0.37 (0.22, 0.61) [**< 0.001**]	116 (42.0) 0.38 (0.23, 0.62) [**< 0.001**]	107 (38.8) 0.48 (0.29, 0.79) [**0.004**]	109 (39.5) 0.98 (0.59, 1.61) [0.923]	89 (32.2) 0.71 (0.42, 1.18) [0.185]	59 (21.4) 0.55 (0.31, 0.96) [**0.036**]	67 (24.3) 0.75 (0.43, 1.31) [0.312]	172 (62.3) 0.45 (0.25, 0.79) [**0.006**]
Any other Asian ethnicity	22 (43.1) 0.22 (0.11, 0.46) [**< 0.001**]	20 (39.2) 0.26 (0.13, 0.53) [**< 0.001**]	20 (39.2) 0.34 (0.17, 0.70) [**0.003**]	22 (43.1) 0.59 (0.29, 1.20) [0.147]	16 (31.4) 0.64 (0.30, 1.33) [0.232]	18 (35.5) 0.76 (0.36, 1.58) [0.456]	13 (25.5) 0.69 (0.31, 1.53) [0.359]	10 (19.6) 0.62 (0.27, 1.42) [0256]	31 (60.8) 0.41 (0.19, 0.89) [**0.024**]
Afro-Caribbean	39 (57.4) 0.30 (0.17, 0.46) [**< 0.001**]	24 (35.3) 0.31 (1.70, 0.55) [**< 0.001**]	32 (47.1) 0.45 (0.25, 0.79) [**0.006**]	20 (29.4) 0.31 (0.17, 0.56) [**< 0.001**]	23 (33.8) 0.53 (0.29, 0.97) [**0.04**]	21 (30.9) 0.51 (0.28, 0.93) [**0.028**]	17 (25.0) 0.64 (033, 1.21) [0.166]	17 (25.0) 0.76 (0.40, 1.46) [0.412]	40 (58.8) 0.27 (0.15, 0.50) [**< 0.001**]
Somali	21 (33.3) 0.16 (0.07, 0.34) [**< 0.001**]	15 (23.8) 0.13 (0.06, 0.28) [**< 0.001**]	3 (4.8) 0.03 (0.01, 0.09) [**< 0.001**]	15 (23.8) 0.24 (0.11, 0.52) [**< 0.001**]	42 (66.7) 3.49 (1.65, 7.38) [**< 0.001**]	3 (4.8) 0.08 (0.02, 0.28) [**< 0.001**]	4 (6.3) 0.13 (0.04, 0.43) [**0.001**]	5 (7.9) 0.23 (0.08, 0.67) [**0.007**]	40 (63.5) 0.48 (0.22, 1.04) [0.064]
Arab	3 (18.8) 0.10 (0.02.0.40) [**< 0.001**]	3 (18.8) 0.13 (0.03, 0.53) [**0.004**]	1 (6.3) 0.05 (0.01, 0.40) [**0.005**]	3 (18.8) 0.24 (0.06, 0.97) [**0.045**]	11 (68.8) 5.76 (1.43, 23.26) [**0.014**]	0 (0) 0 (0.00, i) [0.999]	0 (0) 0.0 (0.00, i) [0.999]	1 (6.3) 0.22 (0.03, 1.80) [0.156]	9 (56.3) 0.47 (0.13, 1.66) [0.241]
Mixed / Other	30 (51.7) 0.28 (0.15, 0.52) [**< 0.001**]	34 (58.6) 1.20 (0.74, 1.95) [0.452]	30 (51.7) 0.60 (0.33, 1.11) [0.103]	25 (43.1) 0.58 (0.31, 1.07) [0.079]	27 (46.6) 1.18 (0.63, 2.18) [0.606]	18 (31.0) 0.56 (0.29, 1.07) [0.130]	22 (37.9) 1.45 (0.77, 2.72) [0.252]	21 (36.2) 1.51 (0.81, 2.83) [0.198]	38 (65.5) 0.43 (0.22, 0.84) [**0.014**]
**Language**									
English	473 (76.0) Ref	375 (60.3) Ref	379 (60.9) Ref	332 (53.4) Ref	295 (47.4) Ref	275 (44.2) Ref	202 (32.5) Ref	184 (29.6) Ref	496 (79.7) Ref
Any other language	186 (49.5) 0.67 (0.41, 1.09) [0.107]	157 (41.8) 1.20 (0.74, 1.95) [0.452]	131 (34.8) 0.95 (0.59, 1.53) [0.830]	134 (35.6) 0.88 (0.54, 1.43) [0.610]	160 (42.6) 0.62 (0.38, 1.01) [0.053]	93 (24.7) 0.68 (0.41, 1.12) [0.130]	75 (19.9) 0.97 (0.56, 1.67) [0.90]	78 (20.7) 0.89 (0.52, 1.53) [0.680]	227 (60.4) 0.57 (0.34, 0.95) [**0.031**]

Awareness of all symptoms, with the exception of ‘blood in stool’ (*p* > 0.05), was statistically significantly lower among Somali adults, compared with White adults (all *p*’s < 0.05). South Asian adults were statistically significantly less likely to be aware of some, but not all, symptoms, when compared with White adults, including: ‘Bleeding from back passage’ (59.4% vs. 80.7%; aOR: 0.44, 95%CI: 0.26, 0.74; *p* = 0.002), ‘Weight loss’ (45.3% vs. 66.1%; aOR: 0.37, 95%CI: 0.22, 0.61; *p* < 0.001), ‘Change in bowel habits’ (39.2% vs. 65.0%; aOR: 0.38, 95%CI:0.23, 0.62; *p* < 0.001), ‘Pain in abdomen’ (38.8% vs. 57.6%; aOR: 0.48, 95%CI: 0.29, 0.79; *p* = 0.004), ‘Tiredness / anaemia’ (21.4% vs. 34.1%; aOR: 0.55, 95%CI: 0.31, 0.96 *p* = 0.036) and ‘Blood in stool’ (62.3% vs. 83.9%; aOR: 0.45, 95%CI: 0.25, 0.79; *p* = 0.006; Table 3).

Similarly, individuals who identified as any other Asian ethnicity were statistically significantly less likely to be aware of several warning signs, when compared with individuals who identified as White British / Irish, including ‘Bleeding from back passage’ (43.1% vs. 80.7%; aOR: 0.22, 95%CI: 0.11, 0.46; *p* < 0.001), ‘Weight loss’ (39.2% vs. 66.1%; aOR: 0.26, 95%CI: 0.13, 0.53; *p* < 0.001), ‘Change in bowel habits’ (39.2% vs. 65.0%; aOR: 0.34, 95%CI: 0.17, 0.70; *p* < 0.003) and ‘Blood in stool’ (60.8% vs. 83.9%; aOR: 0.41, 95%CI: 0.19, 0.89; *p* = 0.024).

Afro-Caribbean adults were also statistically significantly less likely to be aware of multiple warning signs, including: ‘Bleeding from back passage’ (57.4% vs. 80.7%; aOR: 0.30, 95%CI: 0.17, 0.46; *p* < 0.001) ‘Weight loss’ (35.3% vs. 66.1%; aOR: 0.31, 95%CI: 1.70, 0.55; *p* < 0.001), ‘Change in bowel habits’ (47.1% vs. 65.0%; aOR: 0.45, 95%CI: 0.25, 0.79; *p* = 0.006), ‘Pain in abdomen’ (29.4% vs. 57.6%; aOR: 0.31, 95%CI: 0.17, 0.56; *p* < 0.001), ‘Lump in abdomen’ (33.8% vs. 48.3%; aOR: 0.53, 95%CI: 0.29, 0.97; *p =* 0.04), ‘Pain in back passage’ (30.9% vs. 46.6%; aOR: 0.51, 95%CI: 0.28, 0.93; *p* = 0.028) and ‘Blood in stool’ (58.8% vs. 83.9%; aOR: 0.27, 95%CI: 0.15, 0.50; *p* < 0.001).

Arab adults were less likely to be aware that ‘Bleeding from back passage’ (18.8% vs. 80.7%; aOR: 0.10, 95%CI: 0.02, 0.40; *p* < 0.001), ‘Weight loss’ (18.8% vs. 66.1%; aOR: 0.13, 95%CI: 0.03, 0.53;*p* = 0.004), ‘Change in Bowel Habits’ (6.3% vs. 65.0%; aOR: 0.05, 95%CI: 0.001, 0.40; *p* = 0.005), and ‘Pain in Abdomen’ (18.8% vs. 57.6%; aOR: 0.24, 95%CI: 0.06, 0.97; *p* = 0.045) were symptoms, while Mixed / Other adults were less likely to be aware that ‘bleeding from back passage’ (51.7% vs. 80.7%; aOR: 0.28, 95%CI: 0.15, 0.52; *p* < 0.001) and ‘Blood in stool’ (65.5% vs. 83.9%; aOR: 0.43, 95%CI: 0.22, 0.84; *p* = 0.014) were symptoms. There was little evidence of associations for main language, with people whose main language was ‘Any other language’ being less likely, compared with people whose main language was ‘English’, to recognise ‘blood in stool’ (60.4% vs. 79.7%; aOR: 0.57, 95%CI: 0.34, 0.95; *p* = 0.031), only.

With regards to gender, only ‘change in bowel habits’ (46.6% vs. 54.6%; aOR: 1.35, 95%CI: 1.02, 1.78 *p* = 0.360) was associated, with women being statistically significantly more likely than men to know it was a symptom of bowel cancer (Table 3). There was no evidence of an association between any of the warning signs and age.

### Overall risk factor awareness

On average, participants were able to correctly identify 3.99 out of 10 risk factors. After adjusting for co-variates (i.e. gender, age, language), risk factor awareness was statistically significantly lower among Afro-Caribbean (*p* = 0.043) and Somali (*p* < 0.001) participants, compared with White British participants (Table 2). Risk factor awareness did not vary by any other characteristic (Table 2).

### Awareness of individual risk factors

Overall, awareness of individual risk factors was low, with less than 50% of the population being able to correctly recognise any one risk factor. The individual risk factors for which there was the greatest awareness were: ‘low fibre diet’ (*n* = 502; 49.6%), ‘existing bowel condition’ (*n* = 498; 49.2%) and ‘red or processed meat daily’ (*n* = 481; 47.5%; Table 4). The individual risk factors for which there was the lowest awareness were: ‘physical activity weekly’ (*n* = 326; 32.2%), ‘alcohol daily’ (*n* = 285; 28.1%) and ‘having diabetes’ (*n* = 193; 19.1%).

**Table 4:** Predictors of awareness for individual risk factors: results from the binary logistic regression analyses

Symptom	Alcohol Daily n (%) OR (95%CI) [P] (*n* = 926)	Fruit and veg daily n (%) OR (95%CI) [P] (*n* = 923)	Red or processed meat daily n (%) OR (95%CI) [P] (*n* = 932)	Low fibre diet n (%) OR (95%CI) [P] (*n* = 931)	Being overweight n (%) OR (95%CI) [P] (n = 926)	Being over 70 n (%) OR (95%CI) [P] (n = 923)	Close relative CRC n (%) OR (95%CI) [P] (n = 926)	Physical activity weekly n (%) OR (95%CI) [P] (*n* = 928)	Existing bowel condition n (%) OR (95%CI) [P] (*n* = 929)	Having diabetes n (%) OR (95%CI) [P] (*n* = 927)
**Total**										
Total	285 (28.1)	378 (37.3)	481 (47.5)	502 (49.6)	469 (46.3)	398 (39.3)	448 (44.2)	326 (32.2)	498 (49.2)	193 (19.1)
**Gender**										
Male	133 (27.4) Ref	178 (36.7) Ref	233 (48.0) Ref	243 (50.1) Ref	220 (45.4) Ref	201 (41.4) Ref	198 (40.8) Ref	166 (34.2) Ref	224 (46.2) Ref	99 (20.4) Ref
Female	134 (28.2) 1.00 (0.75, 1.34) [1.00]	183 (38.4) 1.04 (0.80, 1.37) [0.754]	223 (46.8) 0.90 (0.69, 1.17) [0.423]	231 (48.5) 0.93 (0.72, 1.21) [0.597]	225 (47.3) 1.04 (0.79, 1.35) [0.800]	174 (36.8) 0.74 (0.56, 0.98) [**0.03**]	230 (48.3) 1.37 (1.04, 1.80) **[0.024]**	143 (30.0) 0.84 (0.63, 1.10) [0.216]	249 (52.3) 1.27 (0.98, 1.65) [0.075]	78 (16.4) 0.76 (0.54, 1.07) [0.110]
**Age (Years)**										
60–65	93 (26.5) Ref	129 (36.8) Ref	158 (45.0) Ref	164 (46.7) Ref	150 (42.7) Ref	128 (36.5) Ref	154 (43.9) Ref	111 (31.6) Ref	170 (48.4) Ref	78 (22.2) Ref
66–69	81 (31.4) 1.21 (0.83, 1.75) [0.326]	111 (43.0) 1.36 (0.96, 1.91) [0.082]	141 (54.7) 1.45 (1.03, 2.04) [**0.031**]	136 (52.7) 1.37 (0.98, 1.92) [0.067]	132 (51.2) 1.42 (1.01, 2.00) [**0.044**]	101 (39.1) 1.10 (0.78, 1.57) [0.587]	130 (50.4) 1.27 (0.90, 1.80) [0.179]	92 (35.7) 1.33 (0.93, 1.90) [0.113]	138 (53.5) 1.28 (0.91, 1.80) [0.152]	46 (17.8) 0.68 (0.44, 1.05) [0.082]
70–75	68 (28.1) 1.15 (0.78, 1.69) [0.487]	88 (36.4) 1.06 (0.74, 1.52) [0.747]	118 (48.8) 1.19 (0.84, 1.68) [0.330]	130 (53.7) 1.55 (1.10, 2.20) [**0.013**]	118 (48.8) 1.34 (0.94, 1.90) [0.100]	109 (45.0) 1.38 (0.96, 1.97) [0.079]	106 (43.8) 0.93 (0.65, 1.33) [0.697]	74 (30.6) 1.08 (0.75, 1.57) [0.682]	119 (49.2) 1.12 (0.791, 1.58) [0.527]	43 (17.8) 0.73 (0.47, 1.13) [0.729]
76+	36 (25.7) 0.89 (0.55, 1.44) [0.639]	44 (31.4) 0.82 (0.53, 1.28) [0.379]	54 (38.6) 0.67 (0.44, 1.03) [0.067]	61 (43.6) 1.01 (0.66, 1.53) [0.979]	60 (42.9) 1.04 (0.67, 1.59) [0.866]	53 (37.9) 0.97 (0.62, 1.50) [0.882]	51 (36.4) 0.63 (0.41, 0.99) [**0.043**]	42 (30.0) 0.99 (0.63, 1.56) [0.969]	62 (44.3) 0.84 (0.55, 1.28) [0.414]	22 (15.7) 0.61 (0.35, 1.98) [0.089]
**Ethnicity**										
White British / Irish	153 (32.4) Ref	186 (39.4) Ref	240 (50.8) Ref	224 (47.5) Ref	243 (51.5) Ref	195 (41.3) Ref	205 (43.4) Ref	147 (31.1) Ref	246 (52.1) Ref	93 (19.7) Ref
South Asian	74 (26.8) 0.60 (0.34, 1.05) [0.074]	95 (34.4) 0.82 (0.45, 1.37) [0.817]	131 (47.5) 0.83 (0.50, 1.35) [0.448]	122 (44.2) 0.85 (0.52, 1.41) [0.535]	131 (47.5) 0.96 (0.58, 1.58) [0.862]	126 (45.7) 1.15 (0.70, 1.90) [0.578]	103 (37.3) 0.62 (0.38, 1.04) [0.068]	89 (32.2) 0.77 (0.45, 1.33) [0.355]	140 (50.7) 1.06 (0.64, 1.75) [0.823]	68 (24.6) 1.05 (0.56, 1.98) [0.883]
Any other Asian ethnicity	17 (33.3) 0.81 (0.38, 1.76) [0.601]	21 (41.2) 1.05 (0.51, 2.16) [0.887]	25 (49.0) 0.80 (0.40, 1.62) [0.534]	23 (45.1) 0.85 (0.42, 1.71) [0.640]	25 (49.0) 1.01 (0.50, 2.07) [0.969]	22 (43.1) 1.05 (0.52, 2.13) [0.894]	19 (37.3) 0.64 (0.31, 1.32) [0.23]	15 (29.4) 0.70 (0.32, 1.52) [0.364]	25 (49.0) 0.94 (0.46, 1.93) [0.874]	10 (19.6) 0.84 (0.34, 2.08) [0.711]
Afro-Caribbean	21 (30.9) 0.80 (0.43, 1.50) [0.493]	21 (30.9) 0.57 (0.31, 1.05) [0.073]	31 (45.6) 0.86 (0.49, 1.52) [0.607]	29 (42.6) 0.67 (0.38, 1.19) [0.173]	23 (33.8) 0.54 (0.30, 0.97) [**0.041**]	21 (30.9) 0.70 (0.38, 1.28) [0.242]	25 (36.8) 0.55 (0.31, 0.98) [**0.044**]	17 (25.0) 0.73 (0.38, 1.39) [0.334]	23 (33.8) 0.46 (0.26, 0.84) [**0.011**]	8 (11.8) 0.42 (0.16, 1.11) [0.080]
Somali	1 (1.6) 0.025 (0.00, 0.20) [**< 0.001**]	25 (40.3) 1.02 (0.49, 2.15) [0.954]	14 (22.2) 0.25 (0.11, 0.55) [**0.001**]	41 (65.1) 2.21 (1.06, 4.61) [**0.035**]	(19.0) 0.254 (0.11, 0.58) [**0.001**]	2 (3.2) 0.04 (0.01, 0.20) [**0.001**]	6 (9.5) 0.12 (0.04, 0.33) [**< 0.001**]	24 (38.1) 0.94 (0.44, 2.01) [0.864]	27 (42.9) 0.82 (0.40, 1.70) [0.594]	2 (3.2) 0.10 (0.02, 0.45) [**0.003**]
Arab	1 (6.3) 0.12 (0.01, 0.98) [**0.047**]	4 (25.0) 0.45 (0.11, 1.82) [0.262]	9 (56.3) 0.36 (0.10, 1.30) [0.118]	2 (12.5) 1.61 (0.47, 5.55) [0.453]	3 (18.8) 0.17 (0.04, 0.85) [**0.03**]	1 (6.3) 0.09 (0.01, 0.76) [**0.026**]	4 (25.0) 0.54 (0.15, 1.98) [0.352]	7 (43.8) 1.66 (0.48, 5.69) [0.420]	5 (31.3) 0.67 (0.19, 2.30) [0.519]	3 (18.8) 0.53 (0.10, 2.79) [0.456]
Mixed / Other	16 (27.6) 0.60 (0.30, 1.21) [0.150]	31 (53.4) 0.99 (0.53, 1.85) [0.981]	32 (55.2) 1.03 (0.56, 1.89) [0.937]	30 (51.7) 1.19 (0.64, 2.21) [0.581]	23 (39.7) 0.97 (0.52, 1.78) [0.908]	26 (44.8) 1.25 (0.67, 2.31) [0.483]	27 (46.6) 0.90 (0.49, 1.68) [0.751]	25 (43.1) 1.41 (0.76, 2.64) [0.278]	27 (46.6) 0.77 (0.42, 1.42) [0.400]	8 (13.8) 0.62 (0.26, 1.50) [0.289]
**Language**										
English	191 (30.7) Ref	317 (51.0) Ref	315 (50.6) Ref	310 (49.8) Ref	240 (38.6) Ref	254 (40.8) Ref	326 (52.4) Ref	189 (30.4) Ref	315 (50.6) Ref	116 (18.6) Ref
Any other language	92 (24.5) 1.41 (0.82, 2.43) [0.217]	161 (42.8) 0.91 (0.56, 1.50) [0.719]	181 (48.1) 1.01 (0.63, 1.62) [0.980]	157 (41.8) 0.81 (0.50, 1.32) [0.400]	134 (35.6) 0.92 (0.52, 1.50) [0.747]	139 (37.0) 1.07 (0.66, 1.73) [0.786]	119 (31.6) 0.66 (0.41, 1.08) [0.099]	134 (35.6) 1.38 (0.82, 2.32) [0.232]	178 (47.3) 0.84 (0.52, 1.36) [0.471]	76 (20.2) 1.31 (0.71, 2.44) [0.389]

As with individual symptoms, Somali participants were statistically significantly more likely to be unaware of most individual risk factors, compared with White British / Irish participants, including: ‘alcohol daily’ (1.6% vs. 32.4%; aOR: 0.03, 95%CIs: 0.20; *p* < 0.001), ‘red or processed meat daily’ (22.2% vs. 50.8%; aOR: 0.25, 95%CI: 011, 0.55; *p* < 0.001), ‘Being overweight’ (19.0% vs. 51.5%; aOR: 0.25, 95%CI: 0.11, 0.58; *p* < 0.001), ‘being over 70’ (3.2% vs. 39.3%; aOR: 0.04, 95%CI: 0.01, 0.20; *p* < 0.001), ‘Close relative CRC’ (9.5% vs. 44.2%; aOR: 0.12, 95%CI: 0.04, 0.33; *p* < 0.001) and ‘having diabetes’ (3.2% vs. 19.7%; aOR: 0.10, 95%CI: 0.02, 0.45; *p* < 0.05; Table 4).

Afro-Caribbean and Arab populations were statistically significantly more ikely to be unaware of several symptoms compared with White British / Irish populations. For example, Afro-Caribbean participants were less likely to recognise ‘Being overweight’ (33.8% vs. 51.5%; aOR:0.54, 95%CI: 0.30, 0.97; *p* = 0.041), ‘close relative with CRC’ (36.8% vs. 43.4%; aOR: 0.55, 95%CI: 0.31, 0.98; *p* = 0.044) and ‘Existing bowel condition’ (33.8% vs. 52.1%; aOR: 0.46, 95%CI: 0.26, 0.84; *p* = 0.011) as risk factors, while Arab participants were less likely to recognise ‘Alcohol daily’ (6.3% vs. 32.4%; aOR: 0.12, 95%CI: 0.01, 0.98; *p* = 0.047), ‘Being overweight’ (18.8% vs. 51.5%; aOR: 0.17, 95%CI: 95%CI: 0.04, 0.85; *p* = 0.03) and ‘Being over 70’ (6.3% vs. 41.3%; aOR: 0.09, 95%CI: 0.01, 0.76*; p* = 0.026).

In addition to ethnic differences, there were gender and age differences for awareness of individual risk factors. For example, women had statiatically significantly higher awareness of ‘Close relative with CRC’ as a risk factor, compared with men (48.3% vs. 40.8%; aOR: 1.37, 95%CI: 1.04, 1.80; *p* = 0.024; Table 4), while adults aged 66–69 were statistically significantly more likely to correctly identify ‘Red or processed meat daily’ (54.7% vs. 45.0%; aOR: 1.45, 95%CI: 1.03, 2.04; *p* = 0.031) and ‘Being overweight as risk factors’ compared to adults aged 60–65 (51.2% vs. 42.7%; aOR: 1.42, 95%CI: 1.01, 2.00 *p* = 0.044), and adults aged 70–75 statistically significantly more likely to correctly identify ‘Low fibre diet’ (53.7% vs. 46.7%; aOR: 1.55, 95%CI: 1.10, 2.20; *p* = 0.013) as a risk factor.

### Screening awareness

Despite nearly all participants being registered with a GP (*n* = 988, 97.5%), and within the eligible age range for bowel cancer screening, only 71.3% (*n* = 722) of participants were aware that a national screening programme for bowel cancer exists. After adjusting for co-variates (i.e. gender, age, language), Afro-Caribbean and Somali adults were statistically significantly less likely to know there is a bowel cancer screening programme compared with White British / Irish participants (awareness was 63, 14 and 81%, respectively; aOR: 0.38, 95%CI: 0.21, 0.70; *p*=0.002; and aOR: 0.05, 95%CI:0.02, 0.12; *p* < 0.001, respectively). There were no statistically significant differences based on gender or main language (all p’s > 0.05; Table 2). Participants over the age of 76 years, however, were statistically significantly less likely to know there is a bowel cancer screening programme than adults aged 60–65 years (awareness was 61 and 69%, respectively; aOR: 0.54, 95%CI: 0.34, 0.85; *p* = 0.009). Conversely, participants aged 70–75 were statistically significantly more likely to know there is a programme than adults aged 60–65 (awareness was 81 and 69%, respectively; aOR: 1.70, 95%CI: 1.08, 2.70; *p* = 0.023).

## Discussion

### Summary of results

This study examined bowel cancer awareness among individuals living within the London Boroughs of Brent, Harrow and Hillingdon. It demonstrates that awareness of CRC symptoms and risk factors is generally low, with individuals correctly identifying (on average) less than 5 out of 9 symptoms and less than 4 out of 10 risk factors. It also demonstrates that awareness of CRC screening is low (especially considering all of the participants were eligible for screening and should have been invited at least once), with only 71.3% of adults correctly identifying that there is a screening programme.

This study also highlights that there are strong associations between ethnicity and CRC screening, risk factor and symptom awareness. It demonstrates that symptom awareness is lower among almost all ethnic minority groups (i.e. those that do not identify as White British or White Irish), and that risk factor and screening awareness are lower for Afro-Caribbean and Somali adults, specifically.

### Comparison with previous literature

Our findings are consistent with those previously described by Power and colleagues (2011), who found that respondents from a non-White ethnic background recognise fewer symptoms and risk factors compared with respondents from a White ethnic background [15]. Our results add to the previous literature, however, by providing more granular information regarding the specific ethnic groups for which risk factor and symptom awareness are independently lower. Our results also add to the literature by investigating associations between ethnicity and bowel cancer screening awareness among groups not included in previous research (e.g. Somali) [8, 15].

The results of our study are consistent with Hirst and colleagues (2018), who previously reported that participation is lower among more ethnically diverse areas, compared with less ethnically diverse areas [8]. Importantly, our study finds that language is not an independent predictor of screening awareness, suggesting language barriers are not responsible for ethnic differences, as previously thought [8]. One possible explanation would be that individuals from specific ethnic groups are less likely to be registered with a general practitioner, which is a prerequisite to receiving screening invitations.

### Implications for future research and practice

This study demonstrates that symptom awareness is universally lower among ethnic minority groups, while risk factor awareness is specifically low for Somali and Afro-Caribbean groups. As such, the results of this study suggest that a broad approach to raising awareness of symptoms among ethnic minority groups is required, while a more specific approach, targeted towards Somali and Afro-Caribbean adults, is required for raising awareness of risk factors. This study also demonstrates that there are specific risk factors and symptoms for which awareness is lower, generally, including ‘Having diabetes’, ‘Alcohol daily’ and ‘Physical activity weekly’, and ‘Bowel not empty’, ‘Tiredness / anaemia’ and ‘Pain in back passage’. As such, the results of this study suggest that health promotion activities seeking to raise awareness in the general population should focus on raising awareness of these specific risk factors and symptoms.

This study also has several important implications for future research. First, further research is needed to understand why awareness of bowel cancer screening is low after adjusting for language, particularly when nearly all of the participants should have received at least one invitation for screening. Second, further research is needed to establish the effectiveness of novel health promotion interventions to increase awareness and reduce inequalities in awareness, and the effects of such interventions on behaviour (screening behaviours, help seeking behaviours, etc.). Finally, further research is needed to assess awareness in other parts of the country, especially where the ethnic composition of the local population is different to that of Brent, Harrow and Hillingdon (for example, areas with large numbers of Chinese adults, who may have lower awareness of specific symptoms and risk factors, which cannot be determinmed from the present analysis, due to small numbers of these individuals).

### Strengths and limitations

This study has several strengths. First, it used a large, ethnically diverse sample, enabling a more nuanced understanding of the relationship between ethnicity and CRC awareness to be developed. Second, it used validated measures for bowel cancer awareness, improving the validity of the study findings. Finally, this study was conducted by community pharmacy healthcare staff, many of whom could speak languages other than English, which meant that it was possible to include non-English speaking participants in the research.

This study also has several limitations. First, there was relatively low participation among CPs. As such, we cannot dismiss the possibility of selection bias. Second, CPs were only required to recruit one participant a day during the study (as a guideline). Here too we cannot dismiss the possibility of selection bias. Third, we did not have an objective measure of screening participation, which would have been more reliable. Fourth, all questionnaires were printed in English. As such, CP staff were required to translate the questionnaire verbally for any non-English language participants they surveyed and, as it was not possible to validate the translations performed, the data for these interviews may be less reliable. Fifth, not all participants were recruited through CP, and so it was not possible to add the pharmacy code as a variable in the analyses (i.e. because it would have led to the exclusion of those participants not recruited through CP). As a result, the possibility of clustering effects and further recruitment bias cannot be discounted. Sixth, the sample size for some ethnic groups was very small (e.g. Arabs). Consequently, the results for these populations should be treated with caution. Finally, this study did not test for interactions between variables (e.g. between ethnicity and gender) and did not include several potentially important confounding variables, including health literacy and years of education. Health literacy, specifically, has been shown to be lower among ethnic minority groups, and may account for the lower awareness scores among some of these groups [21]. Years of education, meanwhile, has been shown to be lower among older adults and is associated with health literacy [22]; as such, the results may not be generalisable to future generations with more years of education.

## Conclusions

This study is the first to demonstrate that there are strong associations between ethnicity and CRC screening, risk factor and symptom awareness. It indicates that there is a special need for effective strategies to raise awareness of specific risk factors and symptoms, particularly among ethnic minority groups, including low physical activity, increased alcohol consumption, diabetes, pain in back passage, anaemia and bowel not feeling empty.

## Supplementary Information

**Additional file 1.** Appendix 1

**Additional file 2.** Appendix 2

## Abbreviations

CP: Community Pharmacy
CRC: Colorectal Cancer
MPC: Middlesex Pharmaceutical Committee
