# Supplementary material for: Ethnic inequalities in older adults bowel cancer awareness: findings from a community survey conducted in an ethnically diverse region in England
Source: BMC Public Health. 2021 Mar 16;21:513. doi: 10.1186/s12889-021-10536-y (PMC7967942; doi:10.1186/s12889-021-10536-y)
Supplement: Supplementary file 1 — Additional file 1. Appendix 1 [file 12889_2021_10536_MOESM1_ESM.pdf]

Putting patients at the HEART of everything we do

ST MARK'S HOSPITAL

NHS
London North West University Healthcare
NHS Trust

Bowel Cancer Awareness Measure

Pharmacy ODS number (F code): ....

Pharmacy Post Code: ....

This survey instrument has been developed by St Mark's Bowel Cancer Screening Centre and University College London. Items on awareness of symptoms and risk factors are based on the generic CAM developed by Cancer Research UK, University College London, Kings College London and Oxford University in 2007-08.

< UCL logo here >

Thank you for taking part in this survey. **Your answers are confidential and totally anonymous.** The information collected is to improve local bowel cancer services. **Only the research team will see information collected**.

1. How old are you?

- [ ] 60 - 65
- [ ] 66 - 69
- [ ] 70 - 75
- [ ] 76 +
- [ ] Prefer not to say

2. What is your gender?

- [ ] Male
- [ ] Female
- [ ] Prefer not to say

3. Which of these best describes your ethnic group?

White

- [ ] White British
- [ ] White Irish
- [ ] Any other White background

Mixed

- [ ] White and Black Caribbean
- [ ] White and Black African
- [ ] White and Asian
- [ ] Any other Mixed background

Asian or Asian British

- [ ] Indian
- [ ] Pakistani
- [ ] Bangladeshi
- [ ] Any other Asian background

Black or Black British

- [ ] Black Caribbean
- [ ] Black African
- [ ] Any other Black background

Chinese/other

- [ ] Chinese
- [ ] Other...
- [ ] Prefer not to say

4. Which is the main language spoken at home?

- [ ] English
- [ ] Urdu
- [ ] Punjabi
- [ ] Gujarati
- [ ] Sylheti
- [ ] Cantonese
- [ ] Other...
- [ ] Perfer not to say

Continue →

5. What are the 1st four digits of your post code? .... ☐ Prefer not to say

6. Have you, your family or close friends had bowel cancer?

	Yes	No	Don't know	Prefer not to say
You	☐	☐	☐	☐
Partner	☐	☐	☐	☐
Close family member	☐	☐	☐	☐
Other family member	☐	☐	☐	☐
Close friend	☐	☐	☐	☐
Other friend	☐	☐	☐	☐

	Yes	No	Don't know	Prefer not to say
**7.** Are you registered with a GP?	☐	☐	☐	☐

8. If ***yes***, where is it located?

- ☐ Brent
- ☐ Hillingdon
- ☐ Harrow
- ☐ Elsewhere
- ☐ Don't know
- ☐ Prefer not to say

Continue →

9. The following may or may not be warning signs for bowel cancer. We are interested in ***your*** opinion:

	Yes	No	Don't know	Prefer not to say
a) Do you think bleeding from your back passage could be a sign of bowel cancer?	☐	☐	☐	☐
b) Do you think persistent pain in your abdomen (tummy) could be a sign of bowel cancer?	☐	☐	☐	☐
c) Do you think a change in bowel habits (diarrhoea, constipation or both) over a period of weeks could be a sign of bowel cancer?	☐	☐	☐	☐
d) Do you think a feeling that your bowel does not completely empty after using the lavatory could be a sign of bowel cancer?	☐	☐	☐	☐
e) Do you think blood in your stools could be a sign of bowel cancer?	☐	☐	☐	☐
f) Do you think pain in your back passage could be a sign of bowel cancer?	☐	☐	☐	☐
g) Do you think a lump in your abdomen (tummy) could be a sign of bowel cancer?	☐	☐	☐	☐
h) Do you think that tiredness/anaemia could be a sign of bowel cancer?	☐	☐	☐	☐
i) Do you think unexplained weight loss could be a sign of bowel cancer?	☐	☐	☐	☐

10. Can you think of any other warning signs for bowel cancer?

....

....

Continue →

11. If you had a symptom that you thought might be a sign of bowel cancer, how soon would you contact your doctor to make an appointment to discuss it?

- Straight away ☐
- Within one week ☐
- Within one month ☐
- After more than a month ☐
- I would not contact my doctor ☐

12. In the next year, who is most likely to develop bowel cancer?

- A 20 year old ☐
- A 40 year old ☐
- A 60 year old ☐
- Bowel cancer is unrelated to age ☐

Continue →

13. The following may or may not increase a person’s chance of developing bowel cancer. How much do you agree that each of these can increase a person’s chance of developing bowel cancer?

	Yes	No	Don’t know	Prefer not to say
a) Drinking more than 1 unit of alcohol a day	☐	☐	☐	☐
b) Eating less than 5 portions of fruit and vegetables a day	☐	☐	☐	☐
c) Eating red or processed meat once a day or more	☐	☐	☐	☐
d) Having a diet low in fibre	☐	☐	☐	☐
e) Being overweight (BMI over 25)	☐	☐	☐	☐
f) Being over 70 years old	☐	☐	☐	☐
g) Having a close relative with bowel cancer	☐	☐	☐	☐
h) Doing less than 30 mins of moderate physical activity 5 times a week	☐	☐	☐	☐
i) Having a bowel disease (e.g. ulcerative colitis, Crohn’s disease)	☐	☐	☐	☐
j) Having diabetes	☐	☐	☐	☐

Continue →

	Yes	No	Don't know
**14.** As far as you are aware, is there an NHS bowel cancer screening programme?	☐	☐	☐

If ***yes***, at what age are people first invited for bowel cancer screening?

....

	Yes	No	Don't know
**15.** Do you know where your local bowel cancer screening center is?	☐	☐	☐

If ***yes***, where do you think your local bowel cancer screening centre is?

....

16. The NHS bowel cancer screening programme is for 60 to 74 year-olds in which people are sent a stool test kit to do at home every two years. This is known as the Faecal Occult Blood (FOB) Test.

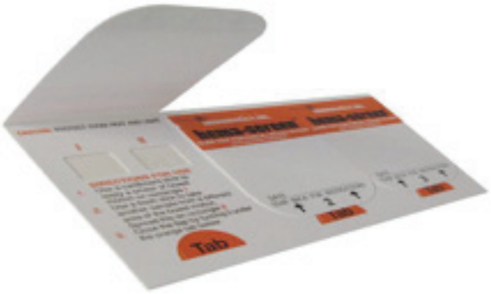

17. Have you ever received a bowel cancer screening stool test kit? (FOBt) test.

☐ Yes

☐ No

skip and take leaflet with freephone number to check your details are in order

☐ Don't know

give leaflet and tell them to call freephone to check

☐ Not eligible

☐ Prefer not to say

skip to end and give leaflet

17b If ***not eligible***, can you please state why?

☐ Prefer not to say

....

Continue →

	Yes	No	
**18.** Have you ever received a stool test kit and not completed it?	☐	☐	If ***no***, skip to question **21** →

19. If ***yes***, which of the following apply?

	Yes	No	Maybe	Prefer not to say
a) I had other more important things to worry about than bowel screening	☐	☐	☐	☐
b) I didn't have any symptoms of bowel cancer	☐	☐	☐	☐
c) I was too busy to complete the bowel screening stool test kit	☐	☐	☐	☐
d) I found it too difficult to complete the bowel cancer screening stool test kit	☐	☐	☐	☐
e) I don't think that I am at risk of developing colorectal cancer	☐	☐	☐	☐
f) I found it too messy to complete the bowel cancer screening stool test kit	☐	☐	☐	☐
g) I found it too embarrassing to complete the bowel cancer screening stool test kit	☐	☐	☐	☐
h) I was too frightened of what the stool test might find	☐	☐	☐	☐
i) I was too afraid of having treatment if it was cancer	☐	☐	☐	☐
j) After thinking about the test, I decided that the risks of taking part outweigh the benefits	☐	☐	☐	☐
k) I didn't want anyone to know that I had completed the bowel cancer screening stool test kit	☐	☐	☐	☐
l) I did not want to have a colonoscopy	☐	☐	☐	☐

20. Is there anything else that would put you off completing a bowel screening stool test kit?

....

....

Continue →

21. ***Women only, up to age 71:***
Would you be willing to receive advice about **breast cancer** screening from your pharmacist?

☐ Yes, definitely ☐ Yes, probably ☐ No, probably not ☐ No, definitely not ☐ Not sure

22. ***Women only, up to age 64:***
Would you be willing to receive advice about **cervical screening** from your pharmacist?

☐ Yes, definitely ☐ Yes, probably ☐ No, probably not ☐ No, definitely not ☐ Not sure

Thank you for completing the questionnaire.

If you have any questions or concerns, please call the screening centre, speak to your GP, or visit the St Marks Bowel Cancer Screening Website.

www.stmarksbowelscreen.co.uk

Honesty Equality Accountability Respect Teamwork
Putting patients at the HEART of everything we do

St MARK'S HOSPITAL

NHS
London North West University Healthcare
NHS Trust

< UCL logo here >
