# Supplementary material for: Ethnic inequalities in older adults bowel cancer awareness: findings from a community survey conducted in an ethnically diverse region in England
Source: BMC Public Health. 2021 Mar 16;21:513. doi: 10.1186/s12889-021-10536-y (PMC7967942; doi:10.1186/s12889-021-10536-y)
Supplement: Supplementary file 2 — Additional file 2. Appendix 2 [file 12889_2021_10536_MOESM2_ESM.docx]

**Appendix 2.**

| **Correlations** | | | | | |
| --- | --- | --- | --- | --- | --- |
|  | | Gender_Dichot | Age | Main_Language_Dichot | Ethnic_group_2 |
| Gender_Dichot | Pearson Correlation | 1 | -.036 | -.008 | .032 |
|  | Sig. (2-tailed) |  | .264 | .799 | .321 |
|  | N | 961 | 957 | 952 | 958 |
| Age | Pearson Correlation | -.036 | 1 | .081^*^ | .024 |
|  | Sig. (2-tailed) | .264 |  | .011 | .446 |
|  | N | 957 | 1003 | 992 | 998 |
| Main_Language_Dichot | Pearson Correlation | -.008 | .081^*^ | 1 | -.398^**^ |
|  | Sig. (2-tailed) | .799 | .011 |  | .000 |
|  | N | 952 | 992 | 998 | 996 |
| Ethnic_group_2 | Pearson Correlation | .032 | .024 | -.398^**^ | 1 |
|  | Sig. (2-tailed) | .321 | .446 | .000 |  |
|  | N | 958 | 998 | 996 | 1004 |
| *. Correlation is significant at the 0.05 level (2-tailed). | | | | | |
| **. Correlation is significant at the 0.01 level (2-tailed). | | | | | |

| **Coefficients^a^** | | | |
| --- | --- | --- | --- |
| Model | | Collinearity Statistics | |
|  |  | Tolerance | VIF |
| 1 | Gender_Dichot | .998 | 1.002 |
|  | Age | .988 | 1.012 |
|  | Main_Language_Dichot | .830 | 1.205 |
|  | Ethnic_group_2 | .837 | 1.194 |
| a. Dependent Variable: Symptoms_Scale | | | |
